# Controversies in Choledochal Malformation in Children: An International Survey among Pediatric Hepatobiliary Surgeons and Gastroenterologists

**DOI:** 10.3390/jcm11041148

**Published:** 2022-02-21

**Authors:** Jan B. F. Hulscher, Joachim F. Kuebler, Janneke M. Bruggink, Mark Davenport, Stefan Scholz, Claus Petersen, Omid Madadi-Sanjani, Nagoud Schukfeh

**Affiliations:** 1 Division of Pediatric Surgery, Department of Surgery, University Medical Center Groningen, 9800RB Groningen, The Netherlands; j.b.f.hulscher@umcg.nl (J.B.F.H.); j.l.m.bruggink@umcg.nl (J.M.B.); 2 European Reference Network RARE-LIVER, 20246 Hamburg, Germany; kuebler.joachim@mh-hannover.de (J.F.K.); petersen.claus@mh-hannover.de (C.P.); madadi-sanjani.omid@mh-hannover.de (O.M.-S.); 3 Department of Pediatric Surgery, Hannover Medical School, 30625 Hannover, Germany; 4 Department of Paediatric Surgery, Kings College Hospital, London SE5 9RS, UK; markdav2@ntlworld.com; 5 Division of Pediatric General and Thoracic Surgery, UPMC Children’s Hospital of Pittsburgh, University of Pittsburgh, Pittsburgh, PA 15224, USA; stefan.scholz@chp.edu

**Keywords:** choledochal malformation, hepaticojejunostomy, Delphi survey, biliary atresia

## Abstract

Background: While congenital choledochal malformation (CCM) is relatively well known within the pediatric surgical and pediatric gastroenterological communities, many controversies and questions remain. Methods: In this paper, we will discuss the results of an international Delphi survey among members of the European Reference Network RARE-LIVER and of the faculty of the Biliary Atresia and Related Diseases (BARD) network to identify the most common practices as well as controversies regarding diagnosis, treatment and follow-up of this still enigmatic disease. Results: Twenty-two individual respondents completed the survey. While there seems to be agreement on the definitions of CCM, preoperative workup, surgical approach and follow-up still vary considerably. The mainstay of treatment remains the removal of the entire extrahepatic biliary tract, clearance of debris both proximally and distally, followed by reconstruction with (according to 86% of respondents) a Roux-en-Y hepaticojejunostomy. Nonetheless, both laparoscopic and robotic-assisted resections are gaining ground with the suggestion that this might be facilitated by concentration of care and resources in specialized centers. However, long-term outcomes are still lacking. Conclusions: As even post-surgical CCM has to be considered as having premalignant potential, follow-up should be well-organized and continued into adulthood. This seems to be lacking in many centers. International cooperation for both benchmarking and research is paramount to improving care for this rare disease.

## 1. Introduction

Congenital choledochal malformations (CCM) are rare anomalies that are characterized by biliary dilatation, usually in the absence of actual biliary tract obstruction. The incidence in the Western world has been estimated in the region of 1:50,000 births, with a marked female preponderance. The etiology is still largely unknown, with both increased pressure and reflux of pancreatic juice due to a long common channel and an anomalous pancreaticobiliary junction as main suspects, although this is far from proven.

CCM can be classified anatomically according to the Todani classification [1] or its derivatives [2], based on morphological appearance and dilatation of the extrahepatic and/or intrahepatic bile ducts. CCM can also be considered a premalignant condition and one systematic review suggested that about 10% of all patients may develop a carcinoma of the biliary tract at some point in life, and usually at a younger age than the normal age of onset of bile duct cancer [3,4]. The long-accepted treatment therefore consists of excision of the entire extrahepatic biliary tree, followed by reconstruction with a Roux-en-Y hepaticojejunostomy.

While this is relatively well known within the pediatric surgical and pediatric gastroenterological communities, there are many questions that remain. In this paper, we will discuss the results of an international survey among pediatric surgeons, hepatobiliary surgeons and pediatric gastroenterologists to identify the most common practice as well as discuss controversies regarding diagnosis, treatment and follow-up of this still enigmatic disease. 

## 2. Materials and Methods

An international online survey was conducted among individual members of the European Reference Network RARE-LIVER as well as individual members of the faculty of the Biliary Atresia and Related Diseases (BARD) network. Questions were drafted (in multiple choice format) by members of the working group biliary malformations (JB, NS, JH, JK), all experienced pediatric hepatobiliary surgeons. The international Delphi survey was performed via an online tool (SurveyMonkey, Survey Monkey Inc., now Momentive Inc., Waterford, NY, USA) and was completed anonymously. Respondents could not be traced back to any participating center. The questions can be found in Supplementary Materials and can be categorized into organization of care, diagnostics and indications for treatment, medical and surgical treatment and follow-up including transition. For all questions, respondents were asked how important they considered the item at hand, ranging from 0–10. We subsequently constructed an ‘importance scale’ based on the median scores of all items and rated accordingly. The outcomes of the questionnaire were subsequently discussed during an online panel discussion during the 2021 online Biliary Atresia and Related Diseases conference (with MD, SS and JH as panelists/moderator).

### Statistics

Continuous and categorical data are quoted as median (range) and percentages, respectively. 

## 3. Results

Twenty-two persons completed the questionnaire anonymously. Out of these, 32% (*n* = 7) stated their country of origin, which was France, Switzerland, Croatia, Denmark, UK, the Netherlands and Germany. 

### 3.1. Organization of Care

CCM was centralized in their country, according to 36% (*n* = 8) of respondents, and 64% (*n* = 14) stated that care was provided by a dedicated pediatric hepatobiliary team, consisting of a pediatric surgeon (*n* = 18; 86%), HPB/liver transplant surgeon (*n* = 8; 38%) and a pediatric hepatologist (*n* = 10; 48%). A nationwide registry was available for children and adults in 14% (*n* = 3) and for children only in 5% (*n* = 1).

### 3.2. Diagnostics

Diagnostic criteria for the presence of a choledochal malformation were a persistent dilatation of the main bile duct (*n* = 20; 91%) and the presence of a common channel ≥10 mm (*n* = 16; 73%). The presence of elevated pancreatic enzymes in bile or a high pressure in the main bile duct were also reported as valuable diagnostic elements in 32% (*n* = 7) and 9% (*n* = 2), respectively. 

All centers performed ultrasound as first choice diagnostic investigation, while an initial CT was performed by none of the centers. Magnetic resonance cholangiopancreatography (MRCP) was performed by 68% (*n* = 15) of the respondents, while 23% (*n* = 5) performed endoscopic retrograde cholangiopancreatography (ERCP). All but one respondent performed a laboratory workup, consisting mainly of cholestatic markers (91%, *n* = 20)), liver function tests (95%, *n* = 21) and coagulation tests (86%, *n* = 19). One third (*n* = 7) of respondents also analyzed tumor markers (e.g., CA19-9).

### 3.3. Indications for Surgery

**Figure 1:**
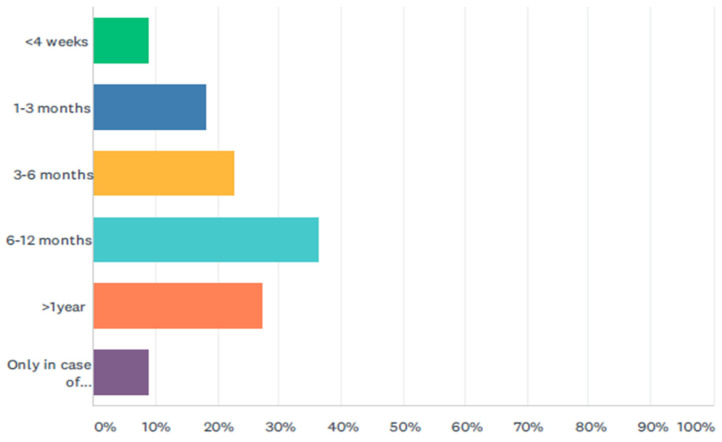
Age at surgery in asymptomatic children.

Ninety-one percent (*n* = 20) of respondents would perform surgery in all children, regardless of the presence of symptoms. Conversely, only two respondents (9%) would operate on symptomatic patients only. The age of surgery in asymptomatic children is depicted in Figure 1. In the specific case of an antenatally detected CCM, most centers tend to operate children before the age of one year, with some 50% operating before six months of age. The presence of complications, i.e., pancreatitis, would lead to postponing surgery for 2 weeks in 27% (*n* = 6) and 6 weeks in 73% (*n* = 16). 

### 3.4. Medical and Surgical Treatment

**Figure 2:**
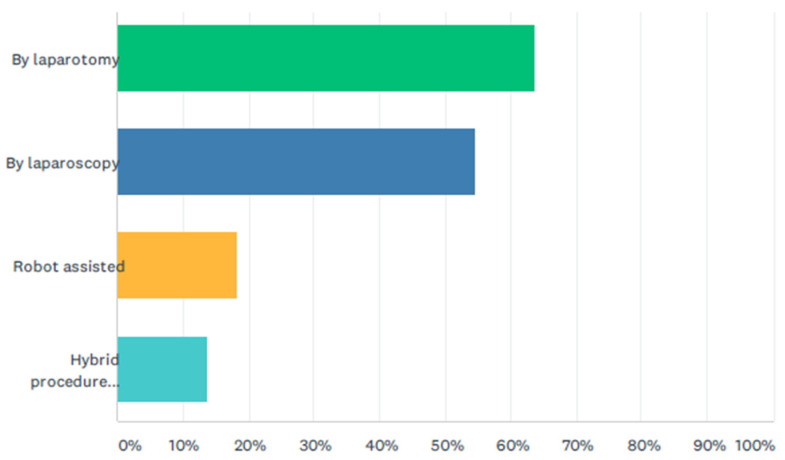
Percentage of choledochal malformations resected by open, laparoscopic and robotic resections.

An open approach was favored by 64% (*n* = 14) of respondents, while laparoscopic and robotic procedures are performed by 55% (*n* = 12) and 18%, (*n* = 4), respectively (Figure 2). Hybrid procedures (laparoscopic dissection and robot reconstruction) are performed in 14% (*n* = 3). Aberrant biliary anatomy and perforated cysts were the most important features leading respondents not to embark on a laparoscopic procedure (26% both). 

**Figure 3:**
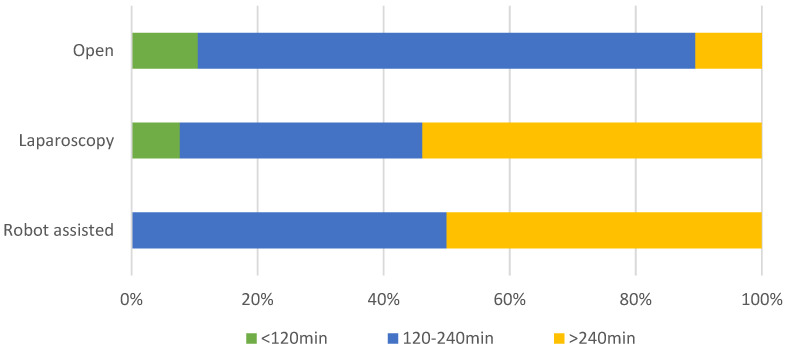
Duration of surgery in open, laparoscopic and robotic procedures.

A peroperative cholangiogram to outline the biliary anatomy (including both proximal and distal dilatations/stenoses) and identify possible ductal stones was performed by 73% (*n* = 16) of respondents. Resection of the extra-hepatic biliary tract followed by Roux-Y hepaticojejunostomy was the preferred approach by 86% (*n* = 19) of surgeons versus resection with hepaticoduodenostomy in 18% (*n* = 4). The length of the Roux loop was between 25–50 cm for 86% of respondents, shorter in 9% and longer in 5%. A total of 86% preferred a retrocolic route. Most (65%) used interrupted sutures for the hepato-enterostomy, with 20% using running sutures and 15% a combination. PDS was the preferred type of suture (80%). Suture size started at 5-0 in 60%, decreasing to 7-0 in 25% of respondents. Stents were avoided by 95% of respondents, with duct size as a possible indication to use a drain. Drains were placed routinely by 70%, never by 10% and on indication only in 25%. A liver biopsy was performed in 55% of cases, evenly divided into wedge or needle. Estimated duration of surgery is shown in Figure 3 with a clear trend toward a longer duration in both laparoscopic and robotic approaches. 

There is a wide variation in the use of peri/postoperative medication. Ursodeoxycholic acid is prescribed by some 60% of respondents, and antibiotic prophylaxis changes widely in duration and type of antibiotic. Nasogastric tubes are placed by 65% of respondents, with 10% mentioning that they only use them in case of a hepaticoduodenostomy. 

### 3.5. Postoperative Follow-Up and Transition of Care

A structural follow-up program is available in 91% of respondents. In two-thirds of cases the pediatric surgeon is the only one following-up on patients, in one-third there is a multidisciplinary team. Follow-up is mainly performed using ultrasound (86%) and laboratory tests (95%), with MRI performed somewhere during follow-up by 20% of respondents. A formal transition program into adulthood was available in 41% (*n* = 9) of hospitals. 

### 3.6. Importance of the Several Items

Table 1 depicts the importance of the different questions as stated by the respondents. The top five (all with a median ≥9.5) of most important topics are the presence of a dedicated team, the use of MRI and ultrasound imaging techniques, centralization of care, life-long follow-up and the length of the Roux-Y loop. Of the first two items, the respondents scored between 7.5 and 10, suggesting a strong agreement. Of the latter three, the ranges were between 1.2 and 10, suggesting a strong discrepancy between the respondents. Respondents considered performing some form of minimally invasive procedure when possible as not important, with a score of 3.3 (range 3.0–10.0). However, there was a large variation between respondents.

## 4. Discussion

This paper gives an overview of several of the most debated questions in the diagnosis and treatment of children with a CCM. It gives an insight into the practices of several internationally acclaimed experts in the field, using both the European Reference Network RARE-LIVER and the Biliary Atresia and Related Diseases network. We do not intend to provide formal guidelines but only want to describe the most common practices of several specialists in the field. We will focus on the topics deemed of most importance by the international respondents. 

### 4.1. Organization of Care

A total 8/22 (36%) respondents stated that care for biliary malformations was centralized in their country. While centralization of care seems beneficial for several surgical procedures, including biliary atresia [5], there is as yet no data available confirming such an effect for choledochal malformation. Given the learning curve of some procedures, especially laparoscopic and robotic CCM resections, which possibly approaches some 35 cases [6], in combination with the extremely low incidence of CCM in the Western world, a reasonable case for centralization could be made [6]. This could easily follow the process for biliary atresia in several countries [7,8,9]. The importance of concentration of care for CCM was acknowledged by >90% of respondents. Of course, depending on the health system and practice environment of individual countries, there are circumstances in which concentration of care might not be feasible nor warranted, which was also remarked on during the panel discussion. This became also apparent from the wide range in importance score: from 3.3 to 10.0. This clearly suggests that centralization is still a highly controversial topic in many healthcare environments. 

Two-thirds of the respondents mentioned that CCM patients were seen and treated by a dedicated pediatric hepatobiliary team, consisting of (among others) a pediatric surgeon (86%), hepato-pancreaticobiliary/liver transplant surgeon (38%) and a pediatric hepatologist (48%). This was considered important by all respondents. Such a team might realistically only be available in high(er) volume centers. These multidisciplinary teams are able to bring collaborative decision making to CCM cases and systemically concentrate clinical experience from multiple specialties on a single complex case. Combined clinics can also improve logistics for the patients. There are drawbacks in the setting up of these teams, such as the time burden for the specialists involved and difficulties with planning, but these might well be overcome by the advantages. Many children’s hospitals are even built around the concept of multidisciplinary teamwork: ‘No other system currently offers so many advantages as the multidisciplinary teams with their pediatricians, surgeons, anesthetists, intensive care specialists, and all the allied health professionals who can add their knowledge to the quality of care’ [10]. This also was clear from the importance score in our survey: a dedicated team scored the highest of all parameters (median 10.0, range 9.4–10.0).

Although considered important by 85%, a nationwide registry was uncommon: for children and adults in 14% and exclusively for children in only 5%. Over the last decade the use of nationwide registries has led to significant improvement in the care for children with rare diseases such as biliary atresia. Similarly, nationwide registries have been paramount in improving care for major surgery for non-rare diseases in adults, such as for colon carcinoma or breast cancer [11]. Most registries aim to improve patient health by improving the quality of patient care. Therefore, monitoring and evaluating patient care are therefore often the primary goals [11]. For rare diseases, with low or very low numbers, national or rather international cooperation is paramount. The initiative to establish an international, prospective online CCM registry has recently been undertaken by the European Reference Network RARE-Liver, in association with the BARD-online registry. This registry could offer an international benchmark, as well as a tool for collaborative research. For all such registries, it is important to define the goal of the registry as well as the governance structure, thereby keeping a keen eye on the balance between the optimal and the feasible [11]. Such a prospective registry might become the new standard for research in rare diseases, alongside the randomized controlled trial which is often difficult to perform when numbers are low [11]. We were therefore surprised to find the nationwide registry not in the top five in the importance score and with a wide range suggesting that several respondents did not consider such a registry as important. 

### 4.2. Diagnostics

Ultrasound was confirmed as the initial diagnostic procedure of choice with an MRCP becoming more widely used in over 60% of centers. ERCP remains less well used at only about 20%. This was in accordance with the importance given to these tests by the respondents. Almost all respondents would order a cholestasis panel/liver function test, and one third of respondents also analyzed tumor markers. However, their importance for children was deemed relatively limited.

Preoperative imaging is aimed at outlining the biliary tree but also identifying stones/debris and delineating a possible pancreaticobiliary maljunction/long common channel. With an up to 80% prevalence of pancreaticobiliary maljunction in CCM patients, an adequate overview of the anatomy of the pancreaticobiliary junction is paramount to be able to safely remove the intrapancreatic bile duct as distally as possible while avoiding pancreatic duct injury [12,13,14]. In addition, identification and removal of ductal stones prior to or during surgery is important. MRCP may miss as many as 40% of PBMs in children, while their detection rate by cholangiography or ERCP approaches 90% [12]. Hukkinen et al. recently demonstrated that the presence of a pancreaticobiliary maljunction is more probable among patients with fusiform CCMs and in those presenting with pancreatitis. If one wants to decrease the risks of ERCP or intraoperative cholangiopancreatography for all patients, patients with fusiform CCM are more likely to benefit from either preoperative ERCP or intraoperative [14]. 

### 4.3. Medical and Surgical Treatment

For virtually all surgeons, the standard treatment is still the removal of the entire extrahepatic biliary tract from the level of the capsule down to the junction with the pancreatic duct, clearance of debris both proximally and distally, followed by reconstruction of continuity of the bilio-digestive tract, thereby diverting bile from pancreatic juices. 

There seems to be an increasing preference toward laparoscopic and robotic resections, especially in Asian centers. These are complex procedures with a significant learning curve, which may be easier to complete in higher volume (i.e., Asian) centers. Two recent meta-analyses demonstrated a similar number of peroperative complications between laparoscopic and open procedures but a significantly shorter hospital stay after laparoscopic procedures [15,16]. Operative time was significantly longer for minimally invasive procedures, which is also found in our survey. This holds true for both laparoscopic and robotic resections. Sun et al. suggested improved long-term outcomes after laparoscopic surgery for CCM, but they almost exclusively included studies from Asia and did not describe any specific outcomes. Recently, Xie et al. described their results with open, laparoscopic and robotic resections [17]. They found that robotic-assisted procedures had similar surgical outcomes as open procedures but were associated with higher medical cost and better cosmetic results [17]. Large multicenter series will be needed to define the role of the different surgical modalities. In the importance score, the use of minimally invasive surgery (when possible) scored surprisingly low (3.3,) but with a wide range (3.0–10.0). This demonstrates the lack of agreement between respondents, on the one hand, but might also point toward seeing minimally invasive surgery in CCM as a means but not as an end. 

In the Dutch cohort, operation before six months and laparoscopic surgery were associated with a higher risk for postoperative complications including anastomotic strictures [18]. Although this was a nationwide study, it represents still a very limited case series. Of all respondents, only one quarter would postpone surgery until at least one year of age in asymptomatic children. This relatively low number is probably due to the fear of symptoms developing and/or the development of liver fibrosis, as well as the fact that CCM can be considered a premalignant disease and limiting exposure is of benefit. On the other hand, evidence of worse outcomes after postponing surgery in asymptomatic children in the literature is very scant. One reason for later surgery is the intent to use the robot, which becomes more feasible in larger children. However, the smallest case operated robotically (published in the Western world) is a child of 5.9 kg [19]. 

In the present survey, hepaticoduodenostomy (HD) is performed by only 20% of respondents. Although we did not specifically ask, we suppose that the respondents favoring HD are mainly laparoscopic surgeons. Hepaticoduodenostomy is technically less demanding, and short-term results are good, without any difference for bile leak, cholangitis or anastomotic strictures. However, a meta-analysis demonstrated that biliary gastritis and gastro-esophageal reflux occur after hepaticoduodenostomy, which is potentially increasing the risk for malignant degeneration [20]. The authors, aware of the controversies, therefore recommend a Roux-en-Y hepaticojejunostomy with a length of some 30 cm, which is in line with the length mentioned by most respondents. Length of the Roux loop was listed in the top five of the importance score. 

What has not been asked in the questionnaire is intraoperative cholangioscopy and the complete wash-out of any biliary debris. Intraoperative cholangioscopy offers the possibility to identify altered mucosa as well as the presence of debris which could (and should) be flushed out. The use of cholangioscopy is not uniform among the authors. Some always perform cholangioscopy, while others perform it on indication only (suspicion of proximal concrements or distal obstruction).

### 4.4. Follow-Up

While follow-up seemed well organized, with a structured follow-up program available for 90% of respondents, transition into adulthood is much less well established, despite the importance of such a transition program as stated by respondents. This was further strengthened by ‘life-long follow-up’ ranking in the top five of the importance score chart. However, the wide range suggests some disagreement between the respondents. In the opinion of the authors, this is especially worrisome due to the premalignant character of choledochal malformations. Two recent reviews of the literature demonstrated a high risk for the development of biliary tract cancer (ranging from 6 to 30%), and this did not completely disappear even after resection of all extra-hepatic bile ducts [3,4] These CCM-associated malignancies occurred at a younger age (around 50 years of age) than sporadic biliary cancers (which occur around 65 years of age) [1]. Therefore, we suggest that follow-up should be performed in centers that specialize in hepatobiliary disorders. This follow-up should include laboratory tests and imaging via ultrasound [2]. Several of the authors also use tumor markers (CA19.9) during follow-up, but the authors realize that evidence thereof is lacking in children [3,21,22]. After transition to adult care, follow-up should be continued by experienced gastroenterologists and hepatologists, with a meticulous handover from pediatric surgeon/hepatologist to the adult colleague.

### 4.5. Limitations

The study is, as are so many questionnaires, hampered by a limited response rate, with only 22 respondents completing the questionnaire. Moreover, the questionnaire might have failed to identify areas of interest. We have not been able to distinguish between adult and pediatric patients nor between physicians caring primarily for adults or children. The study also included experts from different countries with a variety of practice settings and health care systems, which may have influenced their responses. It was also performed within the ERN RARE-LIVER and the BARD community, which might also lead to significant bias (e.g., a preponderance for concentration of care). As the surveys were anonymous, we did not ask for specialty nor center, which we have regretted. We realize that the definitions are rather crude, and the ‘importance score’ might not be scientifically validated. However, we believe that this survey does provide important insights into the hearts and minds of specialists caring for pediatric patients with this rare disease.

## 5. Conclusions

Diagnosis and treatment of congenital choledochal malformations remains challenging. While there seems to be agreement on the definitions of CCM, preoperative workup, surgical approach and follow-up still vary considerably. In the authors opinion, the mainstay of treatment is still the removal of all the entire extrahepatic biliary tract, not just the cyst, clearance of debris both proximally and distally, followed by reconstruction with preferably a Roux-Y hepato-jejunostomy. Although still under some debate, laparoscopy and robotic resections are gaining ground. While the learning curve is fairly long, results are promising in experienced hands. This could imply a benefit for concentration of care to dedicated teams/centers, certainly in the Western world with lower numbers when compared to Asian centers. Such dedicated teams were considered paramount by the respondents. However, long-term outcomes are still to be awaited. CCM has to be considered a premalignant disease, with malignant degeneration occurring in 11% of cases. Follow-up should therefore be well organized and continued into adulthood. This seems to be lacking in many centers. In 2017, the Japanese Study Group on Congenital Biliary Dilatation published a review article with clinical practice guidelines. Due to limited evidence, these guidelines were based on the consensus of experts, using the medical literature for reference [23]. However, international consensus on this topic is still lacking. In our present work, international cooperation for both benchmarking and research is paramount to improving care for this rare disease. First efforts to establish an international registry for quality improvement as well as science have been undertaken by the ERN RARE-Liver in combination with the BARD community, and data entry will be possible in 2022.

## Figures and Tables

**Table 1:** Importance of the topics from the questionnaire based on a scale from 0–10, in order of importance. Data are median (range).

	Median (Range)
Dedicated team	10.0 (9.4–10.0)
Imaging techniques	10.0 (7.5–10.0)
Centralization	9.9 (3.3–10.0)
Life-long follow-up	9.7 (1.2–10.0)
Length of Roux loop	9.5 (2.0–10.0)
Nationwide registry	9.4 (5.0–10.0)
Intraoperative cholangiogram	9.4 (1.0–10.0)
Features for diagnosis	8.5 (2.0–9.9)
Postponing surgery i.c.o. pancreatitis	8.3 (4.5–10.0)
Importance of pancreaticobiliary maljunction for diagnosis	8.3 (6.0–9.9)
Placement of a drain postoperative	8.0 (0.0–9.9)
Obtaining a liver biopsy	8.0 (1.4–9.6)
Delay of surgery until first birthday in asymptomatic patients	7.5 (0.5–10.0)
Leave a cuff of proximal bile duct to suture on	7.2 (1.9–10.0)
Running sutures	4.6 (0.0–9.8)
Nasogastric tube	4.3 (1.0–9.0)
Elevated pancreatic enzymes in bile (for diagnosis)	4.2 (1.0–7.8)
Ursodeoxycholic acid postoperative	4.0 (2.0–9.9)
Minimally invasive surgery (when possible)	3.3 (3.0–10.0)
High pressure in main duct (for diagnosis)	3.2 (1.0–5.0)
Postoperative antibiotic prophylaxis	3.1 (0.1–10.0)
Hepaticoduodenostomy as valid option	1.7 (0.0–10.0)
Running sutures for hepaticojejunostomy	1.5 (0.0–7.7)
Avoiding surgery in asymptomatic patients	0.9 (0.0–3.3)

## Data Availability

The data presented in this study are available on request from the corresponding author. The data are not publicly available due to organisatory reasons.

## Supplementary Materials

The following supporting information can be downloaded at: https://www.mdpi.com/article/10.3390/jcm11041148/s1, Figure S1: Questionnaire Choledochal Malformations.
