# Supplementary material for: Controversies in Choledochal Malformation in Children: An International Survey among Pediatric Hepatobiliary Surgeons and Gastroenterologists
Source: J Clin Med. 2022 Feb 21;11(4):1148. doi: 10.3390/jcm11041148 (PMC8879848; doi:10.3390/jcm11041148)
Supplement: Supplementary file 1 [file jcm-11-01148-s001.zip › jcm-1578919-supplementary.pdf]

Questionnaire Choledochal Malformations

1. How many new choledochal malformations cases are treated per year in your centre?

- **- Type 1:**
- **- Type 2:**
- **- Type 3:**
- **- Type 4:**

2. Do you have a 'dedicated team' in your centre that treats patients with choledochal malformations?

- **- Y/N**

3. Is the care for patients with biliary malformations centralised in your country?

- **-Y/N**
- **- if yes, in which centre(s)?**

4. Do you have a nationwide registry?

- **- yes, for both children en adults**
- **- yes, only for adults**
- **- yes, only for children**
- **- no**

5. Which radiological investigation(s) do you perform when choledochal malformation is suspected? (multiple answers possible)

a. ultrasound

b. CT

c. MRI

d. MRCP

e. ERCP

f. other,

6. Which laboratory tests do you perform at diagnosis?

a. none

b. cholestatic markers

c. liver function test

d. coagulation test

e. tumor markers

7. Main criterium for definite diagnosis of CM

a. Common bilio-pancreatic duct >10mm

b. Persistent dilatation of main bile duct

c. Elevated pancreatic enzymes in bile

d. High pressure in main bile duct

8. In which patients is a surgical procedure performed?

a. all patients

b. only symptomatic patients

c. only patients with high suspicion of malignancy

d. none

9. At what age are asymptomatic children treated at your centre?

a. <4 weeks

b. 1-3 months

c. 3-6 months

d. >6 months

e. Only in case of complications

10. In case of pancreatitis, how long do you postpone surgery:

a. 2 weeks

b. 6 weeks

c. 6 months

11. How is surgery performed at your centre?

a. by laparotomy

b. by laparoscopy

c. robot assisted

12. Which type of surgery is usually performed?

a. exploration, no excision

b. extra-hepatic biliairy tract excision (EHBTE) with E-E anastomosis

c. EHBTE with hepatico-jejunostomy (HJ)

d. cystectomy (without EHBTE)

e. other

13. What is the length of the Roux-Y loop:

a. <25 cm

b. 25-50 cm

c. >50 cm

14. What is the position of the Roux-Y loop;

a. antecolic

b. retrocolic

15. Suturing of hepatoenterostomy is done by

a. Interrupted sutures

b. Running sutures

c. Both (please specify)

Size of suture 5-0 (   ) 6-0 (   ) 7-0 (   )

16. Suturing of entero-enterostomy is done by

a. Interrupted sutures

b. Running sutures

c. Both (please specify)

d. Stapled anastomosis

Size of suture 5-0 (   ) 6-0 (   ) 7-0 (   )

17. Placement of drain

a. yes

b. No

c. Optional

18. Liver biopsy (simultaneously with CM resection)

a. No biopsy

b. Wedge

c. Needle

19. Please complete when applicable:

a. Duration of open CM resection a. < 120 min

b. 120 – 240 min

c. > 240 min

b. Duration of MIS CM resection a. < 120 min

b. 120 – 240 min

c. > 240 min

c. Duration of robotic CM resection a. < 120 min

b. 120 – 240 min

c. > 240 min

20. Peri-operative prophylaxis

a. 1-3 days

b. 1-2 weeks

c. >2 weeks

d. Type of antibiotic:

21. Ursodeoxycholic acid

a. yes

b. no

c. If yes for how long

22. Antibiotics for cholangitis prophylaxis

a. yes

b. no

c. if yes for how long

d. if yes please specify antibiotic

23. Start feeding postoperatively

a. Next day

b. After bowel movement

c. Other indicator, please specify:

21.Tube feeding

a. Yes

b. No

c. Optional

22. Do you have a structural follow-up after resection?

Y/N

23. Which investigations do you perform during follow-up (multiple answers possible)?

a. none

b. laboratory tests

c. by ultrasound

d. by CT

e. by MRI

f. other

24. For how many years do you perform follow up after resection?

25. Does your centre have a transition follow up program for children with choledochal malformations?
